# Supplementary material for: Unveiling the Role of Dissolved Organic Matter on the Hg Phytoavailability in Biochar-Amended Soils
Source: Int J Environ Res Public Health. 2023 Feb 20;20(4):3761. doi: 10.3390/ijerph20043761 (PMC9963283; doi:10.3390/ijerph20043761)
Supplement: Supplementary file 1 [file ijerph-20-03761-s001.zip › ijerph-2213219-supplementary.pdf]

Supporting Information

Unveiling the role of dissolved organic matter on the Hg phytoavailability in biochar-amended soils

Author Names: Wenhao Chen [a,b,1], Zhigang Yu [c,1], Xu Yang [a,b,], Tantan Wang [a,b,], Zihao Li [a,b,], Xin Wen [a,b,], Yubo He [a,b,], Chang Zhang [a,b,] *

Author address:

[a] College of Environmental Science and Engineering, Hunan University, Changsha 410082, China

[b] Key Laboratory of Environmental Biology and Pollution Control (Hunan University), Ministry of Education, Changsha 410082, China

[c] Australian Centre for Water and Environmental Biotechnology, The University of Queensland, St Lucia, QLD 4072, Australia

Analysis of the basic characteristics of biochar and soil

A biochar/water ratio (1:10, g/mL) was used to extract soil DOM with Milli-Q water. pH was measured with a pH electrode. The contents of C and N were determined by an elemental analyzer (Vario EL cube elemental analyzer). The specific surface area of biochar was determined by the N_2-BET method. Morphological analysis of the biochars was performed by Scanning Electron Microscopy with a 15 kV beam (Zeiss Gemini 300). Prior to the analysis, all biochar samples were sputter-coated with gold in a vacuum chamber. After digesting the soil with aqua regia at 95 °C for 2 h, total mercury content was measured using the cold vapor atomic fluorescence spectrometry (CVAFS, Brooks Rand Model, Brooks Rand Labs, Seattle, WA, USA) [1]. Soil organic matter was measured by potassium dichromate oxidation method [2]. Total nitrogen was determined by the macro-Kjeldahl method and total phosphorus was determined by spectrophotometry according to the procedures described in previous studies [3]. Kalium were extracted with 0.1 N ammonium acetate and quantified using a flame atomic absorption spectrometer [4]. Particle size distribution was measured by a laser particle size analyzer (Mastor2000, Malvern Instruments Ltd., UK) in wet mode.

Procedures and parameters of PARAFAC analysis

Fluorescent EEMs were obtained using a fluorescence spectrometer (Hitachi F-

4600 FL; Japan). The fluorescence spectrometer was adjusted to scan at 2400 nm min^{-1} for scanning emission (Em) wavelengths from 250 to 600 nm in increments of 2 nm and excitation (Ex) wavelengths from 200 to 500 nm in increments of 5 nm. The silt widths of both the excitation and emission were set to 5 nm. Prior to PARAFAC analysis, the Raman and Rayleigh scattering of EEM spectra was removed, and the fluorescence intensity was calibrated to Raman units (R.U.) [5,6]. Excitation wavelengths below 240 nm were removed due to deterioration of the signal-to-noise ratio. EEMs of soil DOM were conducted through PARAFAC analysis, performed in MATLAB R2019a using the drEEM Toolbox.

The concentration scores of PARAFAC components were expressed as the maximum fluorescence intensity (F_{max}) of each modelled component and the F_{max} values were reported in Raman units (R.U.) in this study[7,8]. The results of Explained Variation, Core consistency, split-half validation, and visual inspection were used to determine the appropriate components in the DOM[9]. The three fluorescent components were ultimately chosen for this study because it had the second highest Core consistency (55.8%), higher Explained variation (99.45%) and lower Sum of squared residuals (432.66).

Procedures of SFS spectroscopy

Synchronous fluorescence spectra were performed using the same fluorescence spectrometer to record excitation wavelengths from 250-550 nm, a scan rate of 240 nm min-1 for the spectra. The constant offset was chosen to be 60 nm to provide the highest fluorescence intensity, the best resolution and the richest fluorescence signature compared to other offset values[10,11]. To obtain information on changes in the composition of DOM in soil following biochar application, 2D-COS integrated with the synchronous fluorescence spectra was employed by incubation time as the external perturbation. The spectra of each sample used for 2D-COS analysis were first subtracted from the background signal. The 2D-COS analysis was performed using 2D Shige software (Kwansei-Gakuin University, Japan). 2DCOS and hetero-2DCOS maps were plotted using Origin 2022 software.

The amount of using biochar for Hg-contaminated soil remediation

Based on the soil characteristics of the farmland arable layer, it is assumed that the remediated soil depth is 20 cm [12], and the soil density might be 1 g cm^{-3} [13,14]. The biochar is applied at a rate of 5%, i.e., about 5 kg of biochar is required for 100 kg of soil. Then the total amount of biochar to remediate 1 hectare of farmland is about 100 tons (i.e., 100 tons/ha).

Table S1. Basic physical and chemical properties of the study soils

pH	Total mercury ($\mu g\ kg^{-1}$)	Organic matter ($g\ kg^{-1}$)	Total nitrogen ($g\ kg^{-1}$)	Total phosphorus ($g\ kg^{-1}$)	Kalium ($g\ kg^{-1}$)	Sand (%)	Silt (%)	Clay (%)
5.22	234	19.80	1.50	0.48	14.20	48.08	36.65	15.27

Table S2. Basic properties of the biochars.

Biochar	BC300	BC500	BC700
C (%)	49.43	54.00	54.89
N (%)	1.56	1.40	0.88
H (%)	4.406	2.577	1.643
O (%)	24.179	14.589	12.086
C: N	31.68	38.57	62.375
H: C	0.089	0.0477	0.0299
O: C	0.489	0.270	0.220
DOC ($mg\ L^{-1}$)	95.28	18.48	12.73
SSA ($m^2\ g^{-1}$)	1.97	4.43	22.70
pH	7.86	10.58	10.63

Table S3. Descriptions and calculations of UV-vis absorption and fluorescence spectral parameters.

Index	Calculation	Formula parameter	Description
$SUVA_{254}$	$SUVA_{254} = a_{254}$/DOC, $a_{254} = 230.3*A_{254}$	$SUVA_{254}$, a_{254} and A_{254} are the specific UV absorbance, the absorption coefficient and the absorbance at 254 nm wavelength, respectively; DOC is the dissolved organic carbon concentration.	$SUVA_{254}$ is related with DOM aromaticity and humification.
E_2/E_3	$E_2/E_3 = A_{254}/A_{365}$	A_{254} and A_{365} are the absorbance at 254 and 365 nm wavelength, respectively	The E2/E3 ratio is an optical index inversely proportional to the molecular size of aquatic humic substances.
Fluorescence index (*FI*)	$FI = f_{(Ex=370nm,\ Em=470nm)} / f_{(Ex=370nm,\ Em=520nm)}$	f is the fluorescence intensity at a certain wavelength; Ex and Em are excitation and emission wavelength, respectively.	Identify autochthonous (i.e., microbial/algae) and allochthonous (terrestrial) sources in corresponding to FI > 1.9 and FI < 1.4, respectively.
Freshness index (β:α/Frl)	$FrI = f_{(Ex=310;Em=380)}/ f_{(Ex=310;Em=420:435)}$		The freshness index evaluates the proportion of newborn DOM derived from microorganisms.
Humification index (*HIX*)	$HIX = \int_{(Ex=254nm,\ Em=435-480nm)} / \int_{(Ex=254nm,\ Em=300-345nm)}$	$\int$ is the integral of fluorescence intensities in particular wavelength range.	Reflect the degree of DOM humification and the structural complexity.
Biological index (*BIX*)	$BIX = f_{(Ex=310nm,\ Em=380nm)}/ f_{(Ex=310nm,\ Em=430nm)}$		BIX reflects the fresh autochthonous DOM and contribution of protein-like substances. Higher BIX indicates higher biological source and autochthonous feature.

Table S4. The results of PARAFAC analyses of the Explained variance and Core consistency in soil DOM were listed

No. of components	Core consistency (%)	Explained Variation	Sum-of-Squares Error
2	79.763	99.277	574.97
3	55.781	99.45	432.66
4	3.2618	99.502	386.35
5	0.6281	99.537	358.75

Table S5. Spectral characteristics of the excitation and emission maxima of the three components of the EEMs dataset determined by PARAFAC modeling compared to the previously determined sources

Fluorescent Component	Ligand position λEx/Em (nm)	Assignments	Reference λEx/Em (nm)
C1	250,310/425	Soil fulvic acid–like, microbial-transformed	Component 1=<260,320/425[15]<br>Component 1 =<250, 310/414[16]<br>Component 1 =<240, 310/429.5[17]<br>Component 3=<240, 315/425[18]
C2	267,370/480	Terrestrial humic-like substances. Widespread.	Component 2=<260, 360/486[16]<br>Component 2=<250/410[19]<br>Component 2=240,370/480[20]<br>Component 1=<250, 370/475[21]<br>Component 1=336/489[22]
C3	<240,290/375	Protein- and tryptophan-like,microbial-produced	Component4=220–285/368[23]<br>Component 4=250，290/375[24]<br>Component4=285,359/375[25]<br>Component4=220–285/368[23]<br>Component 5==<250, 270/370[26]

Table S6. Spearman's correlation coefficient between phytoavailable Hg contents, soil properties, and DOM characteristics in control and biochar treated soils. The asterisk indicates that the correlation is significant, “*” ($p < 0.05$), “**” ($p < 0.01$).

	P-Hg	pH	DOC	SUVA254	E2/E3	Fl	Frl	BIX	HIX	C1%	C2%	C3%	C1	C2	C3
P-Hg	1.000														
pH	-.671**	1.000													
DOC	-.725**	.571**	1.000												
SUVA254	-.218	.663**	.149	1.000											
E2/E3	.370	-.675**	-.412	-.814**	1.000										
Fl	.392	-.657**	-.328	-.597**	.562**	1.000									
Frl	.529*	-.824**	-.397	-.462*	.427	.768**	1.000								
BIX	.391	-.771**	-.341	-.439	.475*	.744**	.947**	1.000							
HIX	-.132	.254	-.014	-.164	.226	-.277	-.561*	-.540*	1.000						
C1%	.227	-.439*	-.161	-.735**	.662**	.295	.131	.117	.517*	1.000					
C2%	-.556*	.919**	.526*	.734**	-.774**	-.723**	-.815**	-.785**	.218	-.553*	1.000				

C3%	.589**	-.860**	-.517*	-.397	.441	.696**	.932**	.886**	-.583**	.080	-.844**	1.000			
C1	-.489*	.701**	.217	.720**	-.576**	-.451*	-.439	-.403	.030	-.595**	.692**	-.508*	1.000		
C2	-.495*	.786**	.307	.771**	-.687**	-.609**	-.583**	-.564**	.072	-.668**	.833**	-.632**	.953**	1.000	
C3	-.334	.364	.084	.594**	-.462*	-.146	-.023	-.009	-.406	-.759**	.364	-.047	.832**	.756**	1.000

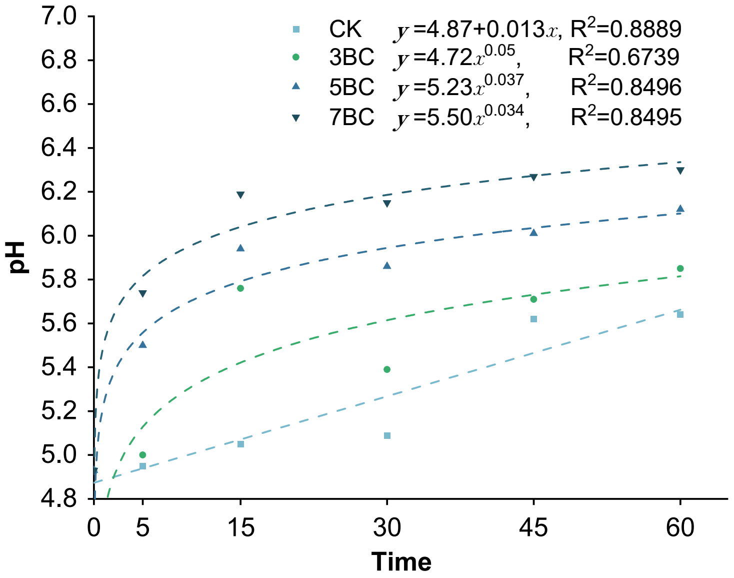

Figure S1. Change in pH over time. The dashed line indicates the fit function. Data at each site are shown as mean values. CK, Control; 3BC, 300 °C biochar treatment; 5BC, 500 °C biochar treatment; 7BC, 700 °C biochar treatment.

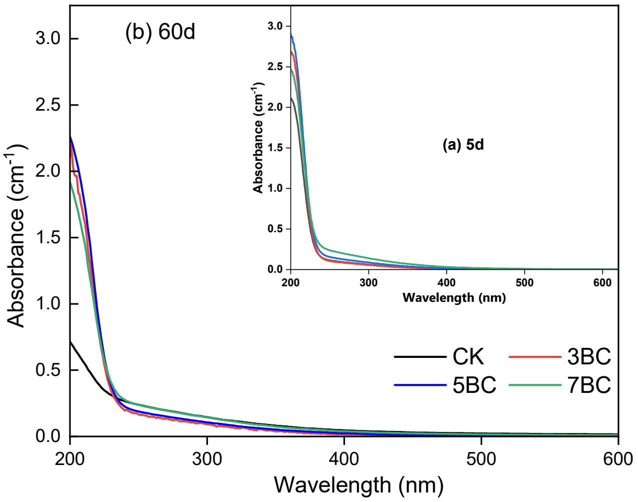

Figure S2. UV-Vis spectral coefficients of DOM in the soil at 5d and 60d for different treatment groups. CK, Control; 3BC, 300 °C biochar treatment; 5BC, 500 °C biochar treatment; 7BC, 700 °C biochar treatment.

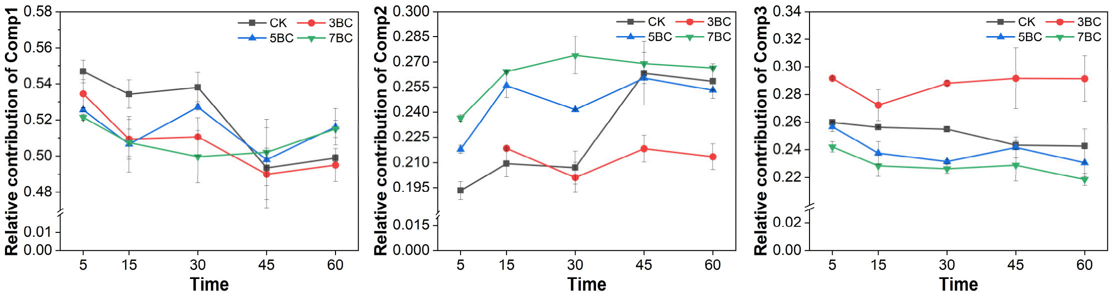

Figure S3. Relative contribution of the three fluorescent components (Comp1 to Comp3) during incubation. The number of replicates was three. CK, Control; 3BC, 300 °C biochar treatment; 5BC, 500 °C biochar treatment; 7BC, 700 °C biochar treatment.

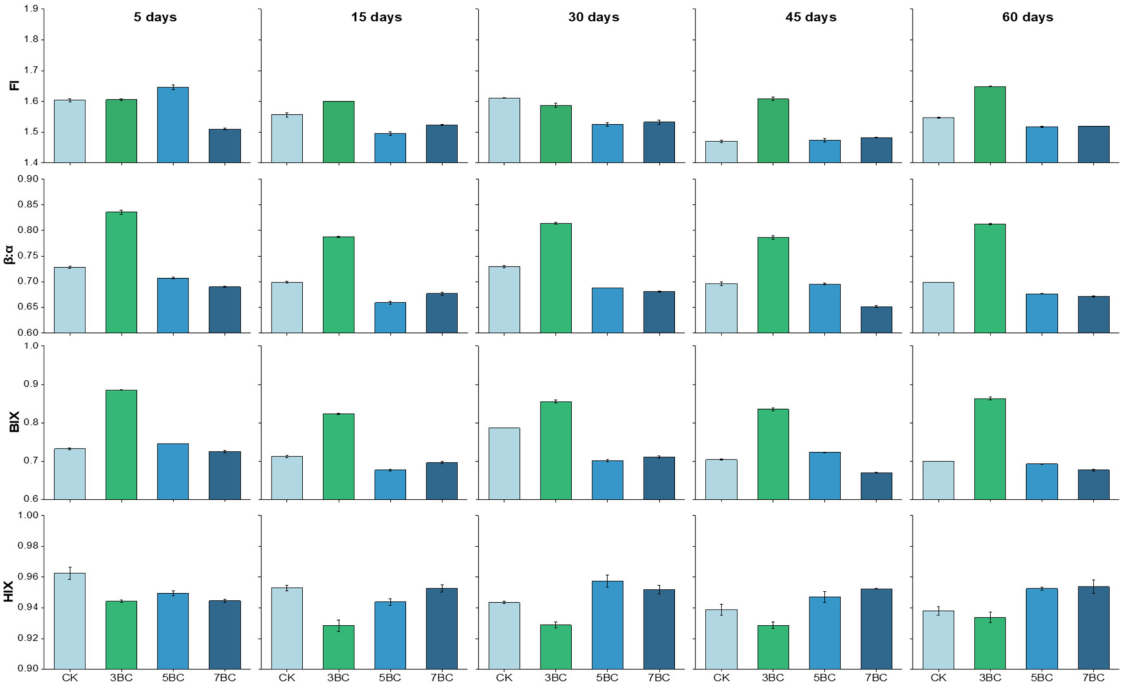

Figure S4. Fluorescence indicators of DOM extracted from soil. FI, Fluorescence index; Frl, freshness index; BIX, biological index; HIX, Humification index. The number of replicates was three. CK, Control; 3BC, 300 °C biochar treatment; 5BC, 500 °C biochar treatment; 7BC, 700 °C biochar treatment.

Reference

1. USEPA. Method 1631 Revision E: Mercury in Water by Oxidation, Purge and Trap, and Cold Vapor Atomic Fluorescence Spectrometry. 2002.
2. Osman, K.S.; Jashimuddin, M.; Haque, S.M.S.; Miah, S. Effect of shifting cultivation on soil physical and chemical properties in Bandarban hill district, Bangladesh. *Journal of Forestry Research* 2013, *24*, 791-795, doi:10.1007/s11676-013-0368-3.
3. Zhao, Y.; Wang, S.; Li, Y.; Liu, J.; Zhuo, Y.; Chen, H.; Wang, J.; Xu, L.; Sun, Z. Extensive

reclamation of saline-sodic soils with flue gas desulfurization gypsum on the Songnen Plain, Northeast China. *Geoderma* 2018, *321*, 52-60, doi:10.1016/j.geoderma.2018.01.033.
4. Gil-Diaz, M.; Luchsinger-Heitmann, A.; Garcia-Gonzalo, P.; Alonso, J.; Lobo, M.C. Selecting efficient methodologies for estimation of As and Hg availability in a brownfield. *Environmental Pollution* 2021, *270*, doi:10.1016/j.envpol.2020.116290.
5. Murphy, K.R.; Stedmon, C.A.; Graeber, D.; Bro, R. Fluorescence spectroscopy and multi-way techniques. PARAFAC. *Analytical Methods* 2013, *5*, 6557-6566, doi:10.1039/c3ay41160e.
6. Lawaetz, A.J.; Stedmon, C.A. Fluorescence Intensity Calibration Using the Raman Scatter Peak of Water. *Applied Spectroscopy* 2009, *63*, 936-940, doi:10.1366/000370209788964548.
7. Bahram, M.; Bro, R.; Stedmon, C.; Afkhami, A. Handling of Rayleigh and Raman scatter for PARAFAC modeling of fluorescence data using interpolation. *Journal of Chemometrics* 2006, *20*, 99-105, doi:10.1002/cem.978.
8. Stedmon, C.A.; Markager, S. Resolving the variability in dissolved organic matter fluorescence in a temperate estuary and its catchment using PARAFAC analysis. *Limnology and Oceanography* 2005, *50*, 686-697, doi:10.4319/lo.2005.50.2.0686.
9. Yang, X.; Li, Z.; Ma, C.; Yang, Z.; Wei, J.; Wang, T.; Wen, X.; Chen, W.; Shi, X.; Zhang, Y.; et al. Microplastics influence on Hg methylation in diverse paddy soils. *Journal of Hazardous Materials* 2022, *423*, doi:10.1016/j.jhazmat.2021.126895.
10. Xing, J.; Xu, G.; Li, G. Analysis of the complexation behaviors of Cu(II) with DOM from sludge-based biochars and agricultural soil: Effect of pyrolysis temperature. *Chemosphere* 2020, *250*, doi:10.1016/j.chemosphere.2020.126184.
11. Chen, W.; Habibul, N.; Liu, X.-Y.; Sheng, G.-P.; Yu, H.-Q. FTIR and Synchronous Fluorescence Heterospectral Two-Dimensional Correlation Analyses on the Binding Characteristics of Copper onto Dissolved Organic Matter. *Environmental Science & Technology* 2015, *49*, 2052-2058, doi:10.1021/es5049495.
12. Kondo, M.; Murty, M.V.R.; Aragones, D.V. Characteristics of root growth and water uptake from soil in upland rice and maize under water stress. *Soil Science and Plant Nutrition* 2000, *46*, 721-732, doi:10.1080/00380768.2000.10409137.
13. Wang, J.; Feng, X.; Anderson, C.W.N.; Qiu, G.; Ping, L.; Bao, Z. Ammonium thiosulphate enhanced phytoextraction from mercury contaminated soil - Results from a greenhouse study. *Journal of Hazardous Materials* 2011, *186*, 119-127, doi:10.1016/j.jhazmat.2010.10.097.
14. Wang, J.; Shaheen, S.M.; Anderson, C.W.N.; Xing, Y.; Liu, S.; Xia, J.; Feng, X.; Rinklebe, J. Nanoactivated Carbon Reduces Mercury Mobility and Uptake by Oryza sativa L: Mechanistic Investigation Using Spectroscopic and Microscopic Techniques. *Environmental Science & Technology* 2020, *54*, 2698-2706, doi:10.1021/acs.est.9b05685.
15. Yamashita, Y.; Kloeppel, B.D.; Knoepp, J.; Zausen, G.L.; Jaffe, R. Effects of Watershed History on Dissolved Organic Matter Characteristics in Headwater Streams. *Ecosystems* 2011, *14*, 1110-1122, doi:10.1007/s10021-011-9469-z.
16. Amaral, V.; Graeber, D.; Calliari, D.; Alonso, C. Strong linkages between DOM optical

properties and main clades of aquatic bacteria. *Limnology and Oceanography* 2016, *61*, 906-918, doi:10.1002/lno.10258.

17. Garcia, R.D.; Reissig, M.; Queimalinos, C.P.; Garcia, P.E.; Dieguez, M.C. Climate-driven terrestrial inputs in ultraoligotrophic mountain streams of Andean Patagonia revealed through chromophoric and fluorescent dissolved organic matter. *Science of the Total Environment* 2015, *521*, 280-292, doi:10.1016/j.scitotenv.2015.03.102.
18. Brym, A.; Paerl, H.W.; Montgomery, M.T.; Handsel, L.T.; Ziervogel, K.; Osburn, C.L. Optical and chemical characterization of base-extracted particulate organic matter in coastal marine environments. *Marine Chemistry* 2014, *162*, 96-113, doi:10.1016/j.marchem.2014.03.006.
19. Wu, H.; Xu, X.; Fu, P.; Cheng, W.; Fu, C. Responses of soil WEOM quantity and quality to freeze-thaw and litter manipulation with contrasting soil water content: A laboratory experiment. *Catena* 2021, *198*, doi:10.1016/j.catena.2020.105058.
20. Kowalczuk, P.; Tilstone, G.H.; Zablocka, M.; Roettgers, R.; Thomas, R. Composition of dissolved organic matter along an Atlantic Meridional Transect from fluorescence spectroscopy and Parallel Factor Analysis. *Marine Chemistry* 2013, *157*, 170-184, doi:10.1016/j.marchem.2013.10.004.
21. Dainard, P.G.; Gueguen, C. Distribution of PARAFAC modeled CDOM components in the North Pacific Ocean, Bering, Chukchi and Beaufort Seas. *Marine Chemistry* 2013, *157*, 216-223, doi:10.1016/j.marchem.2013.10.007.
22. Gold-Bouchot, G.; Polis, S.; Castanon, L.E.; Flores, M.P.; Alsante, A.N.; Thornton, D.C.O. Chromophoric dissolved organic matter (CDOM) in a subtropical estuary (Galveston Bay, USA) and the impact of Hurricane Harvey. *Environmental Science and Pollution Research* 2021, *28*, 53045-53057, doi:10.1007/s11356-021-14509-x.
23. Derrien, M.; Kim, M.-S.; Ock, G.; Hong, S.; Cho, J.; Shin, K.-H.; Hur, J. Estimation of different source contributions to sediment organic matter in an agricultural-forested watershed using end member mixing analyses based on stable isotope ratios and fluorescence spectroscopy. *Science of the Total Environment* 2018, *618*, 569-578, doi:10.1016/j.scitotenv.2017.11.067.
24. Zito, P.; Podgorski, D.C.; Johnson, J.; Chen, H.; Rodgers, R.P.; Guillemette, F.; Kellerman, A.M.; Spencer, R.G.M.; Tarr, M.A. Molecular-Level Composition and Acute Toxicity of Photosolubilized Petrogenic Carbon (vol 53, pg 8235, 2019). *Environmental Science & Technology* 2020, *54*, 5922-5922, doi:10.1021/acs.est.0c01941.
25. Bittar, T.B.; Berger, S.A.; Birsa, L.M.; Walters, T.L.; Thompson, M.E.; Spencer, R.G.M.; Mann, E.L.; Stubbins, A.; Frischer, M.E.; Brandes, J.A. Seasonal dynamics of dissolved, particulate and microbial components of a tidal saltmarsh-dominated estuary under contrasting levels of freshwater discharge. *Estuarine Coastal and Shelf Science* 2016, *182*, 72-85, doi:10.1016/j.ecss.2016.08.046.
26. Williams, C.J.; Yamashita, Y.; Wilson, H.F.; Jaffe, R.; Xenopoulos, M.A. Unraveling the role of land use and microbial activity in shaping dissolved organic matter characteristics in stream ecosystems. *Limnology and Oceanography* 2010, *55*, 1159-1171, doi:10.4319/lo.2010.55.3.1159.
